# Case fatality among people with drug-susceptible TB enrolled in a private health sector TB treatment support program in Bihar, India during the first year of the COVID-19 pandemic

**DOI:** 10.1371/journal.pgph.0003277

**Published:** 2024-09-12

**Authors:** Lena Faust, Ayushi Ranjan, Nita Jha, Madhukar Pai, Sophie Huddart

**Affiliations:** 1 Department of Epidemiology, Biostatistics and Occupational Health, McGill University, Montreal, Canada; 2 McGill International TB Centre, Montreal, Canada; 3 World Health Partners, Patna, India; 4 Department of Epidemiology and Biostatistics, University of California San Francisco, San Francisco, California, United States of America; 5 UCSF Center for Tuberculosis, University of California San Francisco, San Francisco, California, United States of America; Amsterdam Institute for Global Health and Development, NETHERLANDS, KINGDOM OF THE

## Abstract

Experiencing 27% of the global tuberculosis (TB) burden, India’s TB epidemic is the largest in the world. Due to COVID-19-related disruptions to TB programs, India has also seen the largest drop in TB case notifications of any country globally. We estimated case fatality among people treated for TB in India during the pandemic and compared these to pre-pandemic estimates. A random sample of 4,000 adults enrolled in World Health Partners (WHP), a private sector TB treatment support program (enrolling only people with drug-susceptible TB) in Bihar, India in the first year of COVID-19 (Mar 2020-Mar 2021) were contacted via phone to collect information on TB case fatality and other relevant covariates. Inverse probability of selection (IPS) weighting was used to obtain selection-bias-corrected in-treatment and post-treatment case fatality estimates. Covariates associated with (but not necessarily causal of) case fatality were identified by estimating adjusted hazard ratios (HRs) using the Cox proportional hazards model. WHP enrolled 19,826 adult drug-susceptible TB patients in the first year of COVID-19 (Mar 2020 to Mar 2021). Of our random sample of 4,000 patients, n = 2,962 (74.1%) answered the follow-up call. Unweighted and IPS-weighted in-treatment case fatality in the primary analysis were 6.12% (95%CI: 5.31–6.97%) and 6.07% (95%CI: 5.22–6.93%), respectively. Post-treatment case fatality estimates were 0.97% (95%CI: 0.61–1.33%) (unweighted) and 1.27% (95%CI: 0.79–1.79) (IPS-weighted). Our IPS-weighted estimates for in-treatment and post-treatment case fatality were similar to pre-pandemic IPS-weighted estimates (in-treatment: 7.27%, 95%CI: 5.97%- 8.49% and 12 months post-treatment: 1.23, 95%CI: 0.75–1.73). Although not higher than pre-pandemic estimates, the observed case fatality in this private sector cohort of people treated for drug-susceptible TB during COVID-19 in Bihar, India is above the level needed to reach the 2025 and 2030 End TB Strategy targets for reductions in TB deaths, underlining the extent of pandemic-related setbacks to TB elimination.

## Introduction

India experienced 27% of the global TB burden in 2022 [1], making the country’s TB epidemic the largest in the world. Specifically, India had an estimated tuberculosis (TB) incidence of 199 (169–231) cases per 100,000 people in 2022 [2]. Alarmingly, the disruptive impact of the COVID-19 pandemic on TB programs globally [3–6] have been especially felt in India [7], which suffered an 38% reduction in TB case notifications at the beginning of the pandemic [8]; a larger shortfall than any other country [4].

The Indian government declared COVID-19 a ‘notified disaster’ on 14 Mar 2020 [9], and an initial lockdown was enacted across the country on 25 Mar 2020 [10]. Having reported over 45 million COVID-19 cases and over 500,000 associated deaths (although heavily undercounted) [11] as of April 2024 [12], over-burdened health systems and increased barriers to access to care during the pandemic have hindered TB care and prevention, including fewer children receiving the BCG vaccine [7], longer diagnostic delays [13], and fewer patients being started on and successfully completing TB treatment [7]. In addition, pandemic-related disruptions have aggravated existing social determinants of TB in India, including poverty [14] and malnutrition [10].

Even prior to COVID-19, the landscape of TB care in India was complex, with patients navigating fragmented and privatized health systems, resulting in diagnostic delays and loss to follow-up [15]. In particular, India’s large private health sector plays a significant role in TB care (60–85% of TB patients first present for care at private sector facilities) [16], giving rise to unique challenges during COVID-19, including increased out-of-pocket costs being borne by patients [17], and closures of private clinics during lockdown [16].

The quality of TB care provided in the private sector has been extensively evaluated via standardized patient studies, which have found highly variable quality of care [18]. To bridge the gap between private and public health care for TB, Private Provider Support Agencies (PPSAs) have emerged as a key strategy of India’s National TB Elimination Program, with the aim of improving the quality of TB care [19], for example through provision of free diagnosis and treatment, and adherence monitoring. Unfortunately, such PPSA or intermediary agencies have also reported disruptive impacts of the COVID-19 pandemic on their operations, hindering private provider engagement efforts in TB [17].

In this context, COVID-19-related disruptions to TB programs globally, and especially in India, have jeopardized the already fragile progress on TB elimination. In particular, the World Health Organization (WHO) reported that, due to the COVID-19 pandemic, global TB deaths increased in 2020 for the first time since 2005 [4], setting back progress towards WHO End TB goals, which aim to reduce TB-related mortality by 90% by 2030 (compared to 2015 levels) [20]. The government of India has set an even earlier deadline of 2025 for TB elimination in its National Strategic Plan [21]. Given the scale of disruptions to TB services in India, an increase in case fatality among people with TB during the pandemic is likely, making it urgent to assess case fatality in patients diagnosed with TB during COVID-19.

A national-level study examining reported TB mortality in India during the COVID-19 pandemic demonstrates a 15.4% decrease in reported TB deaths in 2020 compared to 2019 [22]. However, given the large decline in TB case detection during COVID-19, it is likely that this decrease in deaths reflects solely a decrease in reporting of cases and outcomes, rather than a true decrease in TB-related deaths. A modelling study predicting additional TB deaths due to missed cases (under-notification) during the pandemic estimated a 19.5% (14.5–24.7%) increase in TB deaths in 2020 [10].

As these studies are limited either by reliance on reported deaths [22], which are likely under-notified, or predictions [10], estimates of case fatality derived from follow-up of TB patient cohorts are needed. A major challenge with such studies is the fact that patients who are unable to be reached for follow-up are more likely to have had poor TB treatment outcomes than patients who are available for follow-up, resulting in biased estimates. A follow-up study of people with drug-susceptible TB enrolled in a PPSA program in Patna, Bihar, India, adjusting for potential selection bias, was carried out by our team prior to the pandemic. This study estimated TB case fatality at 7.27% (95%CI: 5.97%- 8.49%) during treatment, and 1.23 (95%CI: 0.75–1.73) 12 months post-treatment [23]. To examine TB case fatality during COVID-19, we carried out a similar follow-up study in patients diagnosed with drug-susceptible TB in the first year of the COVID-19 pandemic (Mar 2020 –Mar 2021) and enrolled in the same PPSA program.

## Aims

1. To estimate in-treatment and post-treatment case fatality among people diagnosed with drug-susceptible TB in the private sector within the first year of the COVID-19 pandemic in Bihar, India
2. To identify covariates associated with in-treatment and post-treatment case fatality among people diagnosed with drug-susceptible TB in the private sector within the first year of the COVID-19 pandemic in Bihar, India
3. To compare the during-COVID-19 in-treatment and post-treatment case fatality estimates with pre-COVID-19 estimates.

## Methods

### Research ethics

This study was a follow-up survey to ongoing routine, programmatic service delivery by World Health Partners (WHP), a PPSA program in Bihar, India. Ethics approval for the follow-up analysis was therefore obtained from the Bihar state TB program office, and from McGill University (A02-M12-22A). Informed consent was also obtained from each participant via phone call.

### Engagement of communities affected by TB

To ensure this work is relevant to and respectful of the experiences of communities affected by TB, a summary of the research protocol was shared with a panel of five TB survivors and advocates for discussion and incorporation of their feedback into the work.

### Study setting: The WHP PPSA program

The PPSA program, based in Patna, Bihar was established in 2013. Being donor-funded at the outset and operating only in Patna district, the program became government funded and was expanded to operate in 7 other districts (Gaya, Bhojpur, Nalanda, Bhagalpur, Munger, Katihar and Saharsa) in Bihar state as of June 2020.

TB diagnosis and treatment are provided free of charge to patients enrolled in the program, and free testing for co-morbidities (HIV and diabetes) is also available. At the diagnosis stage, the PPSA program’s field officers coordinate sample collection and transport to testing sites, therefore improving linkage between public and private testing facilities and care providers. People diagnosed with drug-resistant TB are referred to the public sector. The program actively maps new care providers to engage, with over 1,400 providers now engaged in the program across the 8 districts in which it operates. Once patients are on TB treatment, treatment adherence support is provided through treatment coordinators who conduct home visits, as well as through call centre staff who conduct biweekly calls to determine treatment adherence and update patient outcomes. The program also provides monthly stipends to patients for food support.

**Fig 1:**
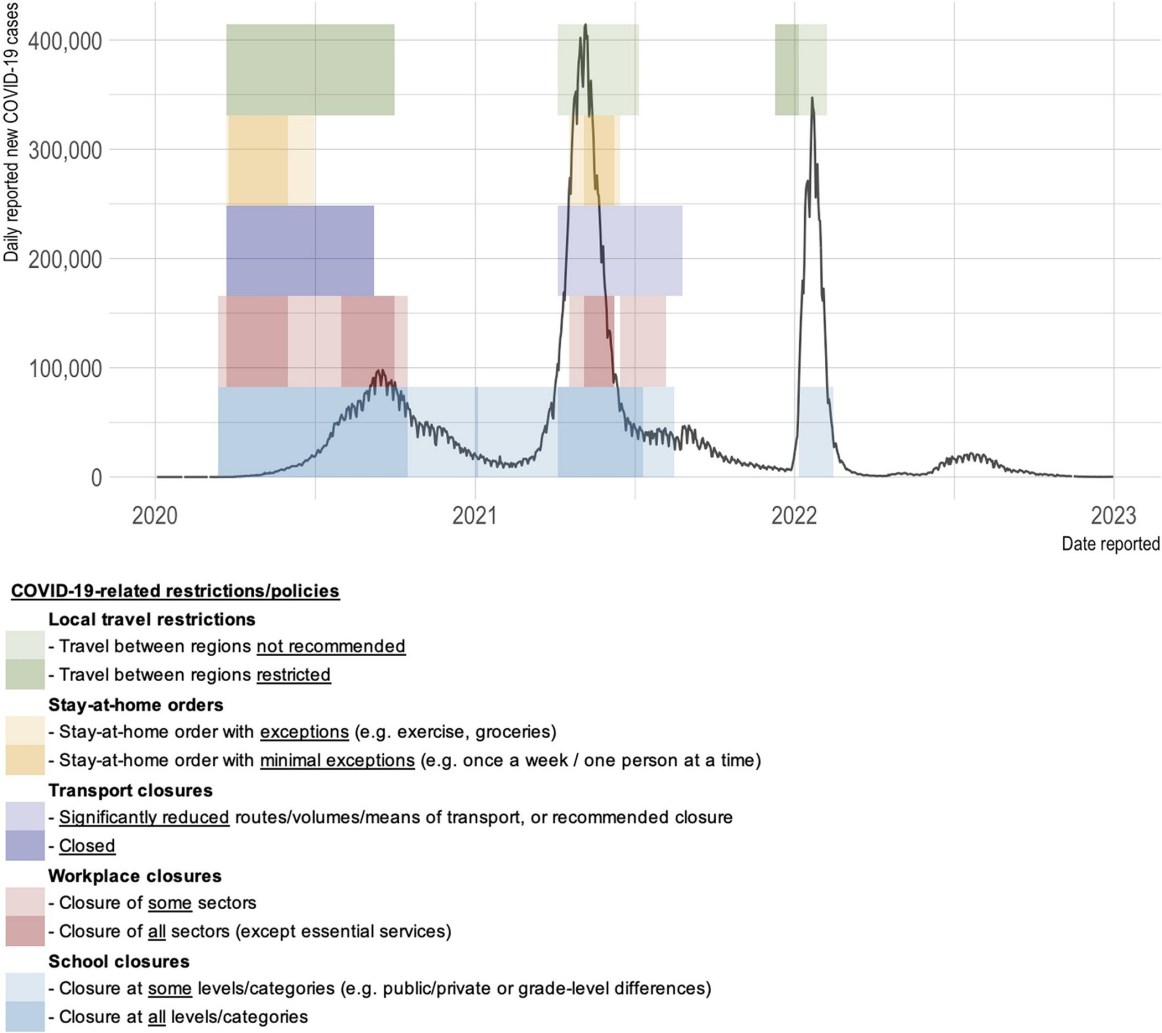
Daily new COVID-19 cases reported in India^24^ and COVID-19-related restrictions [25] in Bihar State (Jan 2020 to Dec 2022). COVID-19 cases are from the WHO COVID-19 database [24]. COVID-19 measures/policies are from the Oxford COVID-19 Government Response Tracker [25].

Like other TB programs, the PPSA program experienced COVID-19-related disruptions to its operations, including difficulties doing household visits and reductions in case notifications due to clinic closures. For context regarding COVID-19 restrictions in the study setting, daily new COVID-19 cases reported in India [24], along with specific COVID-19 related restrictions (e.g. workplace closures, internal movement restrictions, etc.) enacted in Bihar state [25] are shown in Fig 1. In the first year of the pandemic (March 2020 to March 2021) the WHP PPSA program enrolled 24,275 drug-susceptible TB patients, of which 19,826 were adults (≥18 years old). The current study sample (see section below) was drawn from these adults.

### Study sample

A random sample of 4,000 participants was drawn on 15 March 2022 from all adult patients (19,826) in the WHP PPSA program who were diagnosed with drug-susceptible TB in the first year of COVID-19 (March 2020—March 2021).

### Data collection

#### Information available in the PPSA program database

The program records age, sex, site of TB disease (extra-pulmonary/pulmonary), type of case (new or previous TB), district of residence and district of enrolment, diagnosis and enrolment dates, treatment start and end dates, HIV status, diabetes status, treatment adherence (the proportion of doses taken, out of those that should have been taken for the duration of the individual’s treatment, regularly updated through the program’s treatment monitoring calls), and treatment outcome.

#### Primary data collection via phone surveys

Patients in the study sample or their next of kin were contacted via phone to determine the patient’s vital status as well as collect information on covariates not available in the program’s routine database. Phone surveys were conducted between March 2022 and Nov 2022. The study was explained to participants and informed consent was obtained at the outset of each call and recorded on the call sheet. Consenting participants were asked whether they had a COVID-19 test (and if so, its result), they had a diagnosis of COVID-19 by a healthcare provider, they received a COVID-19 vaccine (and if so, how many doses), they smoke currently or have smoked in the past, they had recurrent TB, their self-reported treatment duration (as some patients continue treatment beyond that provided by the program), and, if next of kin answered the call, whether the patient is alive or has died. The dates of these events (where applicable) were also recorded. In addition, due to the lack of indicators of socioeconomic status in the routine PPSA data, participants were also asked about their highest level of education completed. In alignment with the previous PPSA follow-up study [23], patient addresses were also classified as being in “slum” or “non-slum” areas as an indicator of socioeconomic status. All participants or their next of kin were called regardless of WHP-reported treatment outcome, in order to collect data on the variables not available at baseline. Up to three phone calls were attempted for each participant. Home visits to reach patients (after 3 failed calls) were not conducted, given the ongoing pandemic and the fact that they were low yield in the previous study (only 4.7% of respondents [n = 99 out of 2,128] were reached by home visits in the pre-pandemic study) [23]. EpiCollect5 (Oxford Big Data Institute) was used for call data entry.

Definitions of follow-up phases and case fatality are provided in **Box 1**.

**Box 1. Follow-up phases and case fatality definitions**

**Table:** 

**Treatment duration**	In line with the prior study, WHP-reported treatment duration was used unless a participant reported continuing treatment beyond the WHP-recorded treatment duration, in which case the self-reported duration was used.
**Treatment phase** ^**a**^	Time (in months) between treatment start date (WHP-recorded) and treatment completion (WHP-recorded, unless patient reported additional treatment, see above).
**In-treatment case fatality**	A death (from any cause)^b^ occurring during the treatment phase. This aligns with WHO definitions of TB mortality as a death for any reason during or before starting TB treatment [26].
**In-treatment case fatality ratio**	The number of deaths during treatment divided by the number of patients starting the treatment phase.
**Post-treatment phase** ^**a**^	Time (in months) between the end of the treatment phase (see above) and the call date.
**Post-treatment case fatality**	A death (from any cause)^b^ occurring in the post-treatment phase.
**Post-treatment case fatality ratio**	The number of post-treatment deaths divided by the number of patients starting the post-treatment phase.
**End of follow-up**	Call date (for those alive at the time of the call) or death date.

^a^ Deaths could occur in either phase, in which case the date of death is the end date. Censoring only occurred due to end of follow-up (for those alive at the time of the call).

^b^ All-cause mortality includes COVID-19 deaths.

### Sample size

A sample size of 4,000 was selected to allow for a sufficiently precise margin of error (MOE) around the estimate of the weighted CFR. Assuming an in-treatment CFR of 7% based on the prior study [23], we would obtain a margin of error (MOE) of 0.79%. Assuming a 10% loss to follow-up during treatment (based on WHP program experiences) and a post-treatment CFR of 3% [23], the post-treatment CFR would be estimated with a MOE of 0.58%. These MOEs were deemed sufficiently precise to be meaningful for TB programs. In addition, our sample size provided adequate power to detect differences in during-COVID-19 CFRs compared to those in the pre-COVID-19 study [23], with detection of a 2% increase in in-treatment CFR during COVID-19 at 80% power requiring a sample size of n = 2,886 (or n = 1,506 for the post-treatment CFR).

### Data analysis

We conducted a primary and a secondary analysis. The primary analysis included only variables that were available in the pre-COVID-19 follow-up study [23], to facilitate comparability. The secondary analysis included additional variables that were available in the current study but not in the pre-COVID-19 study and that were expected to be associated with case fatality (see *secondary analysis* sub-section).

#### Inverse probability of selection weighting

Based on the pre-COVID-19 PPSA follow-up study [23], we expected a 47% non-response rate. It is possible that patients who could not respond to the survey are more likely to have experienced unfavourable treatment outcomes, including death. Therefore, analyses based only on the subsample responding to the survey are likely to produce biased estimates. Inverse probability of selection (IPS) weights [27] were therefore applied to the observed cohort. This involves specification of a selection model predicting individuals’ probability of response to the phone survey, based on baseline characteristics. These weights are then used to re-weight the sample in subsequent analyses, giving higher weights to individuals less likely to respond to the survey, and thereby correcting for the aforementioned selection bias.

In the primary analysis, covariates included in the selection model were those that were available at baseline, expected to be associated with response to the phone survey as well as the outcome (death), and analogous to those in the selection model of the pre-COVID-19 study [23]. The selection model is shown in **Equation A in S1 Text**. Specifically, covariates included were age, sex, site of disease (pulmonary/extrapulmonary), previous/new TB, slum/non-slum residence, WHP-reported treatment duration (in months), treatment adherence (proportion of doses taken), time since treatment start (months since start of treatment and Nov 2022), and whether the patients’ residence was outside of their district of enrolment in the PPSA program.

Variables in the selection model with missing data included sex (n = 4, 0.1%), new/previous TB (n = 18, 0.4%), slum/non-slum residence (n = 1, 0.03%), WHP-reported treatment duration (n = 8, 0.2%), treatment adherence (n = 74, 1.8%), months since treatment start (n = 8, 0.2%). Given the very low proportion of missing data, a single chained imputation was used to impute missing baseline values [28]. Continuous covariates non-linearly related to survey response (WHP-reported treatment duration and time since treatment start) were modelled flexibly using penalised splines.

#### Observed and unobserved cohorts

**Fig 2:**
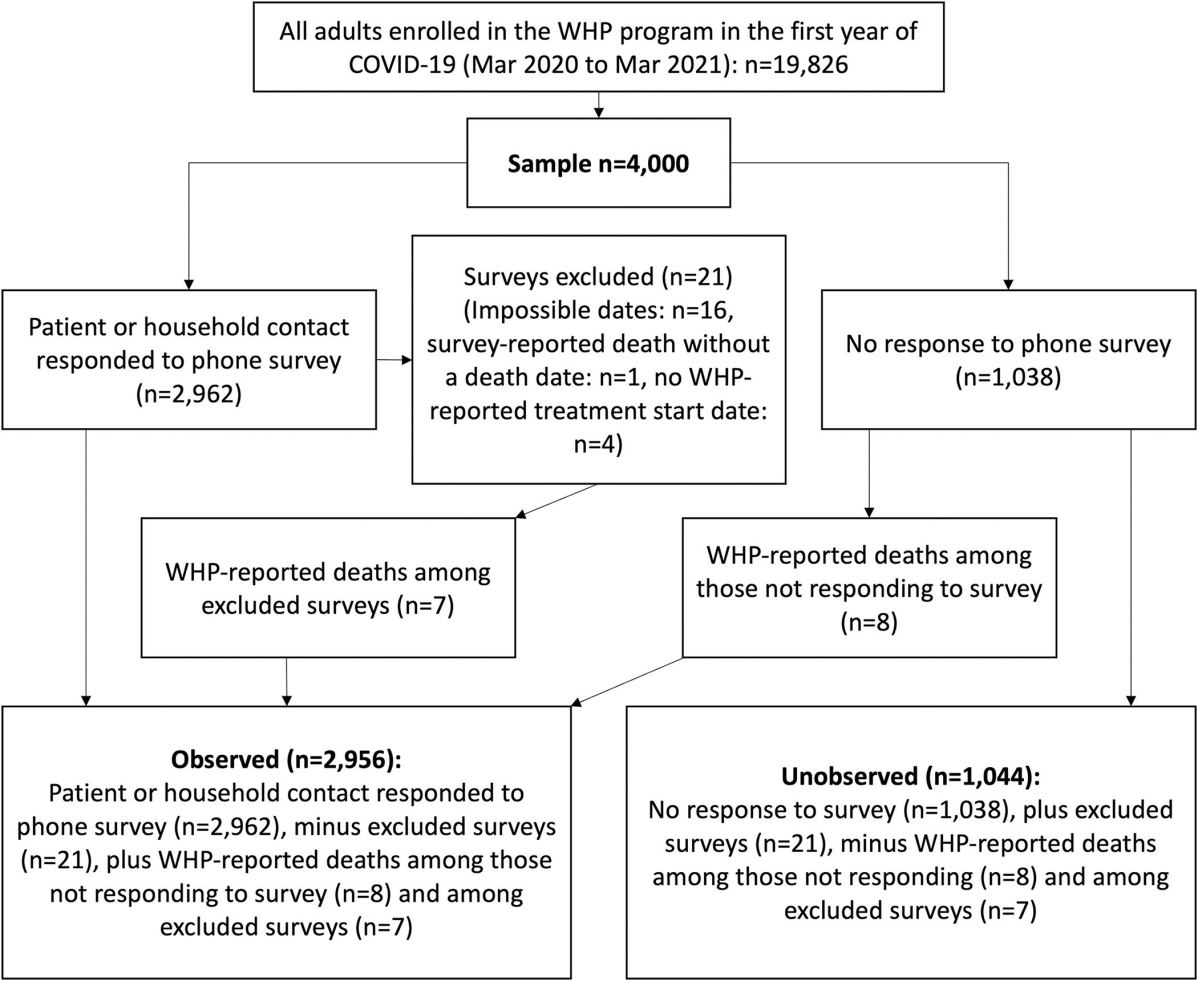
Flowchart of survey responses and inclusion in observed cohort. WHP: World Health Partners (Private Provider Support Agency).

Participants who responded to the survey (and provided valid dates) or were reported by WHP to have died were included in the observed cohort. **Fig 2** describes survey response and inclusion in the observed and unobserved cohorts. The observed cohort (IPS weighted and unweighted) is used in subsequent analyses.

#### Case fatality estimates

In-treatment and post-treatment CFRs were estimated according to definitions in **Box 1.** Both IPS weighted and unweighted CFR estimates (and 95%CIs) were bootstrapped 1,000 times. IPS-weighted Kaplan-Meier curves for in-treatment and post-treatment survival are also shown.

#### Identification of covariates associated with case fatality

Cox proportional hazards models were used to identify covariates associated with case fatality. It should be noted that the goal of this analysis is to identify factors associated with case fatality (rather than causal relationships). Separate models were fit for in-treatment and post-treatment case fatality, using the number of months in the treatment phase or post-treatment phase, respectively, as the time indicator. Results of both IPS weighted and unweighted models are presented, with 95% CIs bootstrapped 1,000 times. Non-linear relationships between continuous covariates and survival were modelled using penalised splines. Where applicable, interaction terms with time (months of treatment or post-treatment, as applicable) were included for terms that violated the proportional hazards assumption (assessed via Schoenfeld residuals). The treatment and post-treatment phase models are shown in **Equations B and C in S1 Text.** Covariates in the primary analysis models included those analogous to those in the pre-COVID-19 study (slum/non-slum, new/previous TB, pulmonary/extrapulmonary TB, age, sex, treatment adherence, and whether the patient resided outside of the district in which they were enrolled into the PPSA program).

#### Secondary analysis

In addition to the variables used in the primary analysis, the selection model in the secondary analysis (**Equation D in S1 Text**) included HIV and diabetes status, as these were available at baseline at the time of the study (captured in routine WHP data, with 10.1 and 11.7% missingness for HIV and diabetes, respectively) and expected to be important factors in non-response, but were not available at the time of the pre-COVID-19 study [23]. In addition, to take into account that the expansion and change in financing of the PPSA program may have resulted in operational changes influencing patient outcomes and likelihood of survey response, an indicator for whether the patient was enrolled before or after the program’s expansion (June 2020) was also included in the selection model. Missing baseline data were again imputed via a single chained imputation and the imputed data used in the selection model. IPS-weighted and unweighted in-treatment and post-treatment CFRs were estimated using the new weights generated by the expanded selection model.

The secondary analysis treatment phase Cox proportional hazards model included HIV and diabetes status, and an additional variable collected in the phone survey–smoking status (0.8% missing), imputed via a single chained imputation. Educational attainment (also collected in the phone survey, to allow more granular indication of socioeconomic status than the slum/non-slum variable) was not included in the final model, as hazards within strata of education were not proportional over time, and introduction of an interaction term with time created convergence issues. The indicator variable for enrolment after the expansion of the PPSA program was also excluded due to convergence issues. COVID-19 co-infection status, although collected in the phone survey, could not be included, as most patients did not report COVID-19 test results (n = 1,843, 62.3%), and of those that did report a result (n = 1,113), only 14 (1.3%) reported a positive test. Due to challenges in accessing COVID-19 testing [29], as well as stigma around COVID-19 in India, especially during the first year of the pandemic [30], it is possible patients were hesitant to disclose getting tested for COVID-19 or receiving a positive test result. Lastly, COVID-19 vaccination status, although also collected in the phone survey, was not included in the model, as 48.6% of participants who reported receiving at least one dose could not provide the date of their first dose, and not accounting for the timing of vaccination poses a risk of introducing immortal time bias [31], making the association between having received the vaccine and survival appear more strongly positive than is accurate.

A secondary analysis for the post-treatment phase Cox proportional hazards model including the additional variables above was not conducted, as the low number of events in the post-treatment phase would not accommodate additional covariates.

#### Sensitivity analysis

Given the difficulties of accessing COVID-19 testing in India, especially early in the pandemic, high missingness was expected for patient-reported COVID-19 test results. A sensitivity analysis using patient-reported diagnosis of COVID-19 by a healthcare provider as an indicator of COVID-19 co-infection (instead of a positive test) was therefore planned, but could not be carried out given that, among the 2,938 people in the observed cohort who responded to the question on healthcare provider diagnoses of COVID-19 (99.4%), none reported having had COVID-19 diagnosed by a healthcare provider.

## Results

Of the 4,000 individuals called, 2,962 (74.1%) answered the phone survey. Valid surveys (n = 2,941) as well as WHP-reported deaths among excluded surveys (n = 7) (see **Fig 2** for exclusion reasons), and WHP-reported deaths among those who did not respond to the survey (n = 8) formed the observed cohort (total observed cohort n = 2,956). **Fig 2** describes survey response and inclusion in the final observed cohort.

Baseline characteristics are shown in **Table 1** for the full cohort (n = 4,000), as well as stratified by observed and unobserved cohort. The full cohort had an average age of 39.0 years (SD: 16.5) and was 61.0% male. Those in the observed cohort were younger, more likely to be female, and more likely to have extrapulmonary TB. Adherence (percentage of doses taken, as determined by WHP treatment monitoring calls) was higher in the observed cohort (81.4%, SD: 35.5) than the unobserved cohort (75.2%, SD: 40.5). While **Table 1** indicates the potential for selection bias due to demographic differences in the observed and unobserved cohorts, **Table 2** subsequently demonstrates that these demographic imbalances have been corrected after IPS weighting.

**Table 1:** Baseline characteristics of people with drug-susceptible TB enrolled in a PPSA program in Bihar within the first year of the COVID-19 pandemic.

Characteristic	Full cohort (n = 4,000)	Observed cohort (n = 2,956)	Unobserved cohort (n = 1,044)
Age (years), mean (SD)	39.0 (16.5)	36.3 (15.1)	46.8 (17.8)
Sex (male), n (%) • Missing, n (%)	2,438 (61.0) 4 (0.1)	1,746 (59.1) 3 (0.1)	692 (66.3) 1 (0.1)
New TB (vs. previous TB), n (%) • Missing, n (%)	3,903 (98.0) 18 (0.5)	2,893 (98.3) 13 (0.4)	1,010 (97.2) 5 (0.5)
Pulmonary TB (vs. extrapulmonary), n (%)	3,202 (80.0)	2,325 (78.7)	877 (84.0)
Adherence (% of doses taken), mean (SD) • Missing, n (%)	79.8 (36.9) 74 (1.9)	81.4 (35.5) 54 (1.8)	75.2 (40.5) 20 (1.9)
Non-slum residence (a), n (%) • Missing, n (%)	3,867 (96.7) 1 (0.03)	2,860 (96.8) 1 (0.03)	1,007 (96.5) 0 (0.0)
Residential district outside of PPSA enrolment district (b), n (%)	852 (21.3)	647 (21.9)	205 (19.6)

(a) Slum/Non-slum indicator used as a proxy for socioeconomic status.

(b) Whether the patient resided in a district outside of the district in which they were enrolled in the PPSA program (which, as of June 2020, operated in 8 districts of Bihar: Patna, Gaya, Bhojpur, Nalanda, Bhagalpur, Munger, Katihar, and Saharsa).

**Table 2:** Covariate balance between full cohort and IPS weighted observed cohort (Mean [Variance]).

Characteristic	Full cohort	Weighted observed cohort
Age (years)	39.00 (273.27)	39.22 (275.01)
Sex (proportion male)^a^	0.61 (0.24)	0.61 (0.24)
Proportion with new TB (vs previous)^a^	0.98 (0.02)	0.98 (0.02)
Proportion with pulmonary TB (vs. extrapulmonary)	0.80 (0.16)	0.80 (0.16)
Adherence (proportion of doses taken)^a^	0.80 (0.14)	0.79 (0.14)
Proportion with non-slum residence (vs. slum residence)^a^	0.97 (0.03)	0.97 (0.03)
WHP-reported months of treatment^a^	6.37 (3.22)	6.35 (3.29)
Proportion with residential district outside of PPSA enrolment district	0.21 (0.17)	0.21 (0.17)

IPS = inverse probability of selection.

^a^ Imputed.

### Primary analysis

In the primary analysis, the selection model (**Equation A in S1 Text**) showed generally good fit, as assessed via a Hosmer-Lemeshow plot (**Fig A in S1 Text**). The IPS weights generated via the selection model and applied to the observed cohort ranged from 1.03 to 5.85, but large weights were uncommon (median: 1.23, 10th and 90th percentiles 1.12 and 1.73, respectively), indicating few highly influential observations. The distribution of IPS weights is shown in **Fig B in S1 Text**. Covariate balance between the full cohort and the weighted observed cohort is shown in **Table 2**. As shown, the IPS weights allowed good balance between the full and observed cohorts, suggesting that the weighted observed sample is representative of the full cohort.

The observed cohort had a mean total follow-up time of 19.3 months (max. 27.0 months), with a mean treatment phase duration of 8.0 months (max. 24.0 months), and a mean post-treatment phase duration of 12.0 months (max. 22.5). The weighted mean treatment and post-treatment phase durations were 8.1 and 12.0 months, respectively.

**Fig 3:**
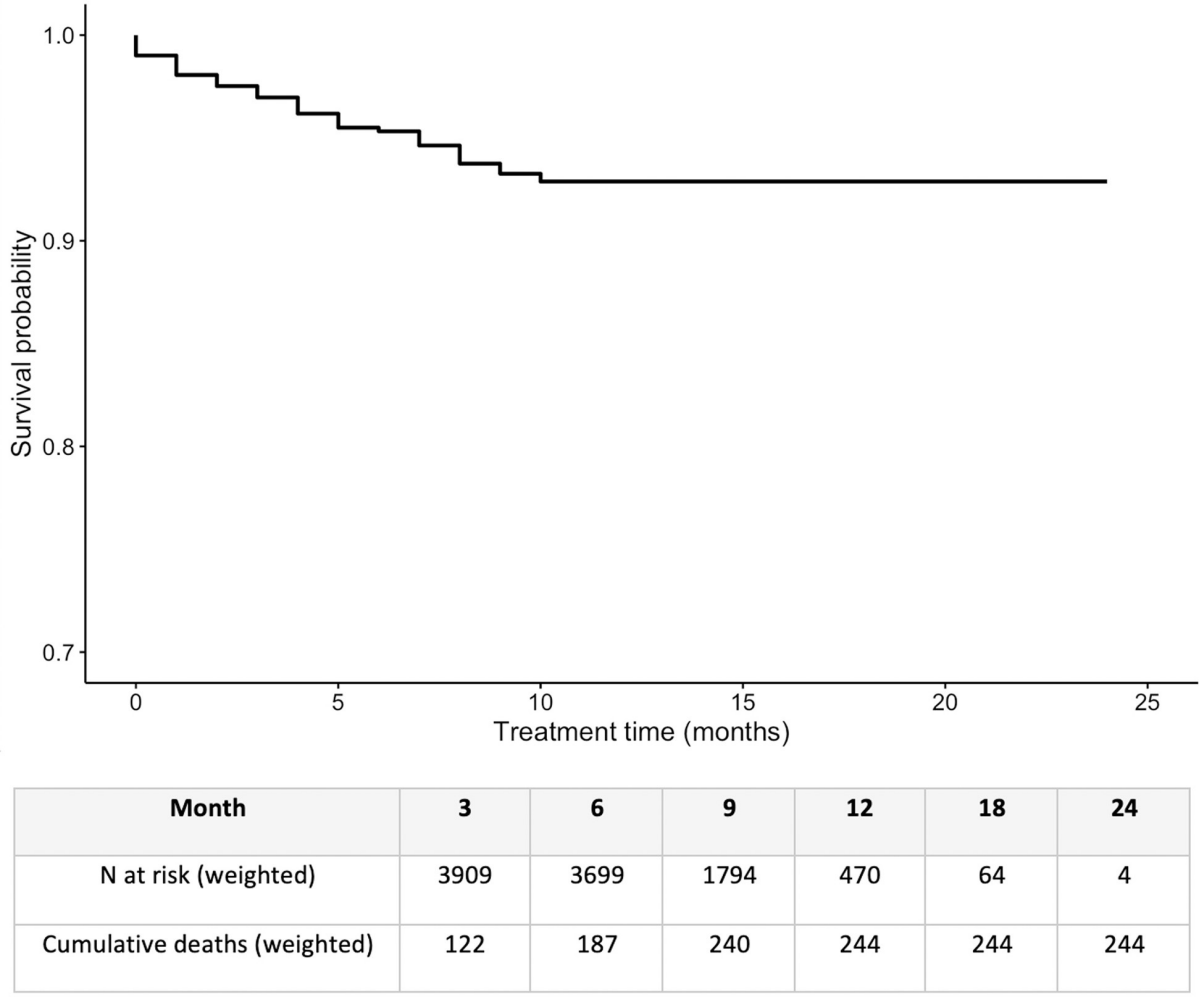
IPS weighted Kaplan-Meier curve and risk table for treatment phase case fatality among people with drug-susceptible TB in Bihar, India, in the first year of the COVID-19 pandemic.

The IPS-weighted Kaplan-Meier curve for treatment phase survival is shown in **Fig 3**. Most deaths occurred within the first 9 months of the treatment phase. IPS-weighted and unweighted treatment phase cumulative hazards at 3, 6, 9, 12, 18, and 24 months are shown in **Table A in S1 Text.** Overall unweighted treatment phase case fatality was 6.12% (95%CI: 5.31–6.97%) (IPS weighted CFR: 6.07%, 95%CI: 5.22–6.93%). The unweighted treatment phase CFR from the current study (during COVID-19) is higher than the unweighted CFR estimated in the pre-COVID-19 follow-up study of patients enrolled in the same PPSA program (4.15%, 95%CI: 3.56%-4.83%), however, the IPS weighted during COVID-19 estimate is similar to that of the pre-COVID-19 study (CFR = 7.27%, 95%CI: 5.97–8.49%). Nonetheless, the CFR estimates in the current study remain above the target CFR (5%) required to reach the End TB Strategy goals [32].

**Fig 4:**
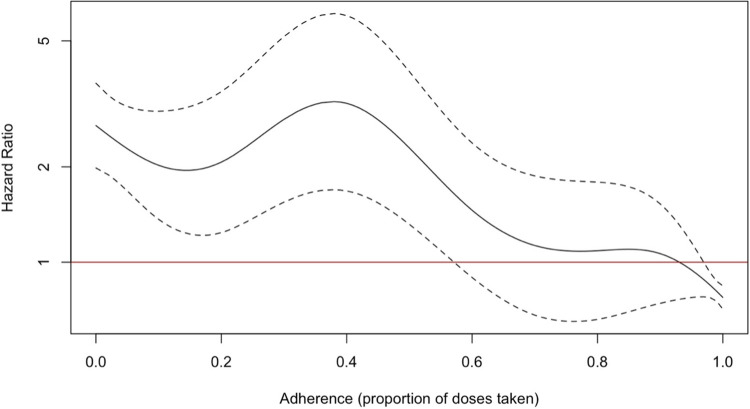
Non-linear relationship between TB treatment adherence proportion and hazard of in-treatment death. IPS-weighted hazard ratios for treatment phase case fatality vs. treatment adherence (imputed), holding other covariates constant. Modelled using penalised splines (df = 4). Dashed line = 95%CI of hazard ratios. Red line = null hypothesis hazard ratio (1.0). IPS = inverse probability of selection.

The analysis model, identifying covariates associated with treatment phase fatality are shown in **Table 3**. The non-linear relationship between treatment adherence proportion and survival is shown in **Fig 4**. Only age was significantly associated with in-treatment case-fatality in both the unweighted and weighted models (unweighted model HR for each one-year increase in age: 1.04, 95%CI: 1.03–1.05, for 10-year increase in age: 1.49, 95%CI: 1.37–1.64, weighted model HR for one-year increase in age: 1.03, 95%CI: 1.02–1.05, for 10-year increase in age: 1.39, 95%CI: 1.27–1.55). Patients residing outside of their PPSA enrolment district had significantly higher case fatality in the unweighted model (HR: 1.50, 95%CI: 1.05–2.13), but not in the weighted model (HR: 1.43, 95%CI: 0.97–2.13). This is similar to findings in the pre-COVID-19 study, where only age was significantly associated with survival (in both the unweighted and weighted models). The relationship between treatment adherence and survival was non-linear (**Fig 4**) and was modelled flexibly. 60% or lower adherence significantly increased the hazard of in-treatment death.

**Table 3:** Covariates associated with treatment phase case fatality among people with drug-susceptible TB in Bihar, India, in the first year of the COVID-19 pandemic.

	Unweighted			IPS weighted		
	HR	95%CI		HR	95%CI	
Slum residence (Ref: Non-slum residence)	1.01	0.25	1.98	1.09	0.26	2.22
Previous TB (Ref: New TB)	1.49	0.51	3.04	1.57	0.45	3.44
Pulmonary TB (Ref: Extrapulmonary TB)	1.25	0.87	1.90	1.29	0.88	2.02
Age (per year)	1.04	1.03	1.05	1.03	1.02	1.05
Sex, Male (Ref: Female)	0.99	0.73	1.39	0.96	0.69	1.36
District of residence outside PPSA enrolment district (yes/no)	1.50	1.05	2.13	1.43	0.97	2.13

IPS = inverse probability of selection.

**Fig 5:**
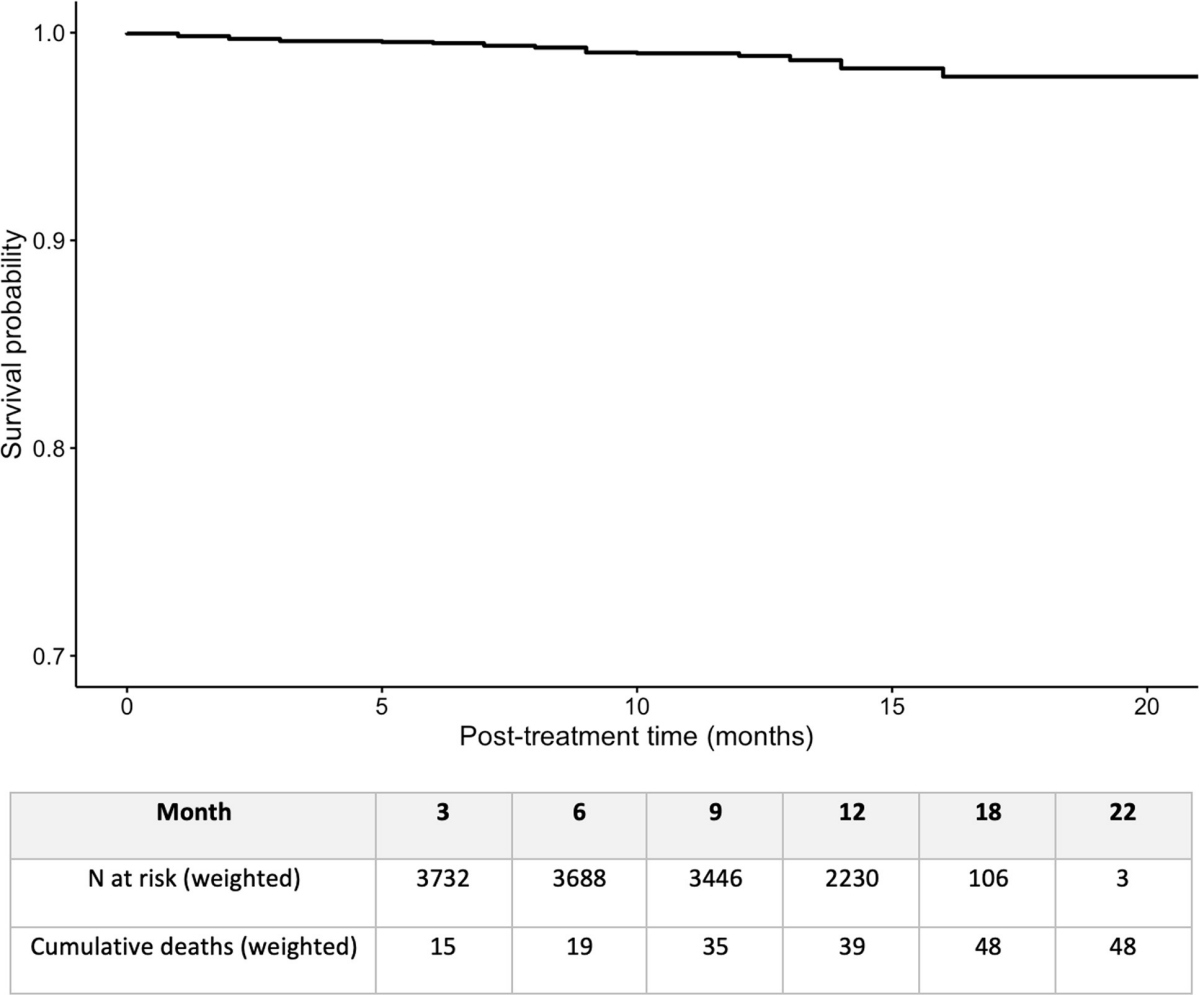
IPS weighted Kaplan-Meier curve and risk table for post-treatment case fatality among people with drug-susceptible TB in Bihar, India, in the first year of the COVID-19 pandemic.

The IPS-weighted Kaplan-Meier curve for post-treatment phase survival is shown in **Fig 5**. IPS-weighted and unweighted post-treatment cumulative hazards at 3, 6, 9, 12, 18, and 22 months are shown in **Table B in S1 Text.** Post-treatment case fatality was 0.97% (95%CI: 0.61–1.33%) (unweighted) and 1.27% (95%CI: 0.79–1.79) (weighted). Weighted post-treatment case fatality in the current study was similar to weighted pre-COVID-19 post-treatment case fatality estimates at 12 months post-treatment (1.34, 95%CI 0.90–1.99 unweighted and 1.23, 95%CI: 0.75–1.73 weighted) [23]. Although the weighted 24-month post-treatment case fatality in the pre-COVID-19 study was 3.32% (95%CI: 2.36–4.42%) [23], the 12-month time point allows a more appropriate comparison given the mean follow-up time of 12.0 in the post-treatment phase in the current study.

Hazard ratios for post-treatment phase fatality are shown in **Table 4**. No significant associations with post-treatment case fatality were found, possibly due to the low number of events (deaths) in the post-treatment phase.

**Table 4:** Covariates associated with post-treatment case fatality among people with drug-susceptible TB in Bihar, India, in the first year of the COVID-19 pandemic^(a)^.

	unweighted			weighted		
	HR	95%CI		HR	95%CI	
Slum residence (Ref: Non-slum residence)	1.15	0.00	4.53	0.93	0.00	3.74
Previous TB (Ref: New TB)	2.57	0.00	9.56	1.71	0.00	6.15
Pulmonary TB (Ref: Extrapulmonary TB)	1.01	0.45	3.83	0.87	0.38	3.42
Sex, Male (Ref: Female)	1.38	0.62	3.25	1.47	0.64	3.43
Adherence (proportion of doses taken) (b)	0.71	0.29	3.08	0.68	0.26	3.31
District of residence outside PPSA enrolment district (yes/no)	0.84	0.17	2.15	1.06	0.20	2.77

(a) An interaction term between age (years) and post-treatment time (months) was included in the model due to violation of the proportional hazards assumption. Its values are reported in S1 Text.

(b) Splines not used as non-linear term for adherence was non-significant.

### Secondary analysis

In the secondary analysis, the expanded selection model (**Equation D in S1 Text**) showed good fit (**Fig C in S1 Text**), and the IPS weights applied to the observed cohort using this expanded model ranged from 1.02 to 6.07, with large weights being uncommon (median: 1.23, 10th and 90th percentiles 1.12 and 1.74, respectively). Weights again produced good balance between the full cohort and the re-weighted observed cohort (**Table C in S1 Text**). The weighted mean treatment and post-treatment phase durations remained similar to the primary analysis (8.1 and 12.0 months, respectively). The weighted in-treatment CFR was 6.09 (95%CI: 5.25–6.96), similar to the weighted in-treatment CFR in the primary analysis. The weighted post-treatment CFR was 1.27% (95%CI: 0.80–1.81), again similar to the primary analysis.

Results of the expanded Cox proportional hazards model identifying covariates associated with in-treatment case fatality are shown in **Table D in S1 Text**. In the weighted model, only age was significantly associated with survival, with older individuals more likely to die during treatment (HR for one-year increase in age: 1.04, 95%CI: 1.03–1.05, for 10-year increase in age: 1.42, 95%CI: 1.29–1.58). Counterintuitively, patients who reported current or past smoking were significantly less likely to die during treatment (HR: 0.31, 95%CI: 0.16–0.51). These covariates were also significantly associated with survival in the unweighted model. The relationship between treatment adherence and survival was non-linear (**Fig D in S1 Text**), with adherence below approximately 60% significantly associated with a higher hazard of in-treatment case fatality.

Primary and secondary analysis in-treatment and post-treatment CFRs are compared in **Table 5**. Although estimates in primary and secondary analyses are similar, differences are most likely attributable to the inclusion of additional variables in the selection model in the secondary analysis, resulting in differences in IPS weights. Given the similarity between primary and secondary analysis estimates and that a bias analysis on a cohort of patients enrolled in the same PPSA program [33] showed that inclusion of additional confounders in the selection model had minimal effect on estimates, we consider our primary analysis results to be reliable (see discussion section).

**Table 5:** Comparison of IPS weighted case fatality ratio estimates from the primary and secondary analyses.

	Primary analysis	Secondary analysis
**In-treatment CFR**	6.07% (95%CI: 5.22–6.93%)	6.09 (95%CI: 5.25–6.96)
**Post-treatment CFR**	1.27% (95%CI: 0.79–1.79)	1.27% (95%CI: 0.80–1.81)

IPS = inverse probability of selection.

## Discussion

Our findings indicate a selection-bias-corrected in-treatment CFR of 6.07% (95%CI: 5.22–6.93%) among people diagnosed with drug-susceptible TB in the private health sector during the COVID-19 pandemic and enrolled in a PPSA program in Bihar, India. This is similar to the pre-COVID-19 study’s bias-corrected CFR of 7.27% (95%CI: 5.97–8.49%) [23]. Although disruptions to TB programs during the pandemic likely increased case fatality among people with TB, a possible explanation for the fact that we did not observe a higher CFR in the current study (compared to pre-COVID-19 estimates) is that due to COVID-19-related barriers to care-seeking for TB [4–6], more severely ill patients were less likely to reach care (i.e., be diagnosed and enrolled in the PPSA program). This delayed care seeking may have caused more pre-treatment deaths, which would not be reflected in our study. This suggests that overall TB case fatality, taking into account pre-treatment deaths, may be higher than the treatment and post-treatment fatality estimated in this work.

it is also possible that due to barriers to accessing care during COVID-19, patients that *did* reach care presented with more advanced disease [34]. Due to the absence of data on delays from symptom onset to treatment initiation, or disease severity at treatment initiation, however, we were unable to assess whether people initiating treatment during COVID-19 were more severely ill at baseline. The fact that our fatality estimates are similar to those estimated pre-COVID-19 study suggests that, at least from treatment initiation onwards, COVID-19 has not substantially altered treatment outcomes at this stage of the care cascade. Research to understand and mitigate damage to earlier steps in the cascade, likely more heavily impact by COVID-19, is needed.

Regardless, our during-COVID-19 CFR is above the CFR target of 5% articulated in the WHO’s South-East Asia region-specific strategic plan for reaching the End TB goals [32]. This underlines that we are not on track to global TB elimination goals, which are especially urgent in a country that shoulders more than one quarter of the total burden of TB cases worldwide [1].

In addition, given that our sample was drawn from patients enrolled in a PPSA program which offers a variety of services beyond the standard of care (adherence support, food support, provider engagement), it is likely that case fatality in the general population in India is even higher than estimated in this study. In particular, it is possible that the disruptive impacts of the COVID-19 pandemic felt in public TB programs (barriers to accessing care) were attenuated in this population, who were more closely followed throughout treatment (regular adherence support calls by PPSA staff). Due to receiving food support, this population may also have been shielded to some extent from the economic impacts of COVID-19 on households, which would otherwise have exacerbated poor TB outcomes (due to malnutrition, etc.) [10].

Our findings are also subject to other important limitations. A major limitation is survey non-response, given that individuals who could not respond to the survey may be more likely to have experienced unfavourable TB treatment outcomes, which would bias estimates that are based only on the observed sample. For this reason, IPS weights were applied to re-weight the sample based on their probability of response (conditional on baseline covariates), thereby assigning higher weights to individuals less likely to respond and adjusting for selection bias. Despite this effort to reduce bias, residual confounding in the selection model remains a limitation. In particular, given that information on socioeconomic status (such as income) is not collected at baseline in routine WHP data, this could not be included in the selection model, and the slum / non-slum residence indicator was therefore used as an imperfect proxy of socioeconomic status. The similarity between weighted and unweighted in-treatment CFRs however indicates low selection bias in our study, possibly due to the relatively high survey response rate of 74.1% (the pre-COVID-19 PPSA follow-up study had a response rate of 53.2%). It is also possible that patient follow-up within the PPSA program has improved since the previous study, resulting in fewer in-treatment deaths going unreported by the program.

A further limitation relating to survey non-response is that home visits (to reach participants who could not be reached by phone) were not carried out in our study, given the safety concerns and logistical challenges of doing home visits during COVID-19. It is possible that the pandemic altered phone access, especially in light of increased poverty during the pandemic [14], and the fact that families may have lost members of working age, meaning that we may have been less likely to reach the most vulnerable participants. However, considering our high response rate to the phone survey (74.1%) and the fact that home visits were low-yield in the pre-pandemic study (only 4.7% of participants reached via home visit) [23], not conducting home visits is not expected to have significantly impacted our comparison to pre-pandemic estimates. Further, our use of IPS weighting addressed the potential selection bias induced by non-response.

It should also be noted that the CFRs presented here represent all-cause mortality, meaning that it is possible that a considerable percentage of deaths in the current study (as opposed to the pre-COVID-19 study) were due to COVID-19, especially given the higher risk of mortality among patients co-infected with COVID-19 and TB [35]. However, the use of all-cause mortality in this study is consistent with the WHO definition of TB deaths, which considers a death from any cause during TB treatment to be a TB death [26]. Further, although all-cause mortality as measured in this study does not differentiate between COVID-19 deaths and TB deaths, we do not believe that mortality reporting by next of kin would differ substantially based on cause of death. Although stigma around COVID-19 has been reported in India [30,36], TB and its symptoms were already stigmatised in this population even prior to COVID-19 [37], so the additional stigma of COVID-19 is not expected to have substantially influenced reporting of fatality compared to in the pre-COVID-19 study. Moreover, while COVID-19 is a serious disease, its fatality rate is dwarfed (particularly in this relatively young population) by that of TB, which had a 12.3% during-treatment case fatality ratio globally in 2022 [1], and leaves survivors with a standardized mortality ratio of 2·91 (95%CI: 2·21–3·84) [38].

A further limitation is that, by nature of the survey-based design, we relied on patient self-report for many important variables. This limited the reliability of some data collected. For example, we could not assess the impact of COVID-19 co-infection on case fatality (in the secondary analysis) due to low reporting of positive COVID-19 test results. The barriers to accessing COVID-19 testing [29] as well as stigma associated with COVID-19 in India make it likely that many patients who had COVID-19 were not tested [36] or were uncomfortable reporting their test results [30]. Although we planned to address the problem of low COVID-19 testing rates through a sensitivity analysis, using patient-reported diagnosis of COVID-19 by a healthcare provider, no patients reported such a diagnosis. The limited reliability of self-reported data is also evident in the fact that we could not assess TB recurrence given that only 8 patients reported valid recurrence dates (within the post-treatment phase).

Further, we could not investigate COVID-19 vaccination status as a factor in fatality, as almost half (48.6%) of all participants who reported receiving at least one dose of the vaccine could not provide the date their first dose was received. Not being able to account for the timing of vaccination poses a risk of introducing immortal time bias [31], as COVID-19 vaccination was rolled out in India on 16 January 2021 [39], and the majority (70.7%) of our sample was enrolled in the WHP program before this date. It is therefore likely that the fact that individuals enrolled earlier had to survive long enough to have received a vaccine would make the positive association between COVID-19 vaccination and survival appear stronger than is accurate.

In addition, the counterintuitive protectiveness of smoking against case fatality observed in the secondary analysis is most likely due to the fact that smoking status was unavailable at baseline, and that self-reported smoking during follow-up calls was unreliable, possibly leading to residual confounding. Given the stigma associated with smoking among people with TB, it is possible that patients who successfully quit were more likely to report past smoking. However, a bias analysis [33] simulating smoking, HIV and malnutrition prevalence among the pre-COVID-19 PPSA cohort found that their inclusion in the selection model did not substantially change estimates, suggesting that our primary analysis remains robust even without inclusion of these variables.

Another limitation of the survey-based design is that, although analysis of both the pre- and during-pandemic data in the same model would have allowed a clearer comparison of outcomes in the two cohorts, it would have required re-contacting participants of the pre-pandemic study for consent to use their data for a new purpose, which was judged as infeasible given the time elapsed since enrollment of the pre-pandemic cohort would make it difficult to reach a high number of patients from this cohort.

Lastly, other than COVID-19, the PPSA program also underwent significant changes during the study period, changing from donor to government funding, and expanding operations to 7 additional districts. This reduces comparability between the pre-COVID-19 estimates in the prior follow-up study (at which time the program operated only in one district and was donor-funded) and our during-COVID-19 estimates. In addition, it is possible that differences in treatment outcomes during COVID-19 are not only the result of the pandemic but also the result of the programmatic and operational changes, introducing secular trends that are not attributable to COVID-19 disruptions alone.

Nevertheless, our results show fairly high TB case fatality, which is concerning, especially given India’s accelerated timeline of reaching the End TB goals 5 years before the global target. Regaining lost progress on TB elimination as programs recover from COVID-19 will require improving TB case-detection and reducing loss to follow-up along the TB care cascade, including through treatment adherence support [40], as is being done in the PPSA program. PPSAs and their work engaging TB care providers to bridge gaps in fragmented health systems are therefore key to supporting TB elimination.

## Supporting information

S1 Text (DOCX)
