# Supplementary material for: Case fatality among people with drug-susceptible TB enrolled in a private health sector TB treatment support program in Bihar, India during the first year of the COVID-19 pandemic
Source: PLOS Glob Public Health. 2024 Sep 12;4(9):e0003277. doi: 10.1371/journal.pgph.0003277 (PMC11392330; doi:10.1371/journal.pgph.0003277)

**Supplementary material for Case fatality among people with drug-susceptible TB enrolled in a private health sector TB treatment support program in Bihar, India during the first year of the COVID-19 pandemic**

Faust L,^1,2^ Ranjan A,^3^ Jha N,^3^ Pai M,^1,2^ Huddart S^4,5^

1. Department of Epidemiology, Biostatistics and Occupational Health, McGill University, Montreal, Canada
2. McGill International TB Centre, Montreal, Canada
3. World Health Partners, Patna, India
4. Department of Epidemiology and Biostatistics, University of California San Francisco, San Francisco, USA
5. UCSF Center for Tuberculosis, University of California San Francisco, San Francisco, USA

Section 1: Primary analysis

**Equation A.** Selection model for prediction of probability of response based on baseline covariates, used to generate inverse probability of selection weights

$logit\left( p\left( R=1 | X \right) \right)=\beta_{0}+\beta_{1}X_{age}+\beta_{2}X_{sex}+ \beta_{3}X_{SiteofDisease}+ \beta_{4}X_{PreviousOrNew}+ \beta_{5}X_{Residence}+ \beta_{6}X_{OutsideEnrolDist}+spline\left( tx\_duration \right){+ \beta}_{7}X_{adherence}+spline\left( since\_tx \right)$

*Where: R = 1 = Responded to phone survey, SiteofDisease = Extrapulmonary/pulmonary TB, Previous/new = previous or new TB case, Residence = Slum/non-slum residence, OutsideEnrolDist = Residential district outside PPSA enrolment district, tx_duration = WHP-reported months of treatment duration, adherence = proportion of doses taken, since_tx = months since start of treatment and Nov 2022 (last month of phone survey)’*

**Fig A.** Hosmer-Lemeshow plot of selection model fit (observed vs predicted values):

**
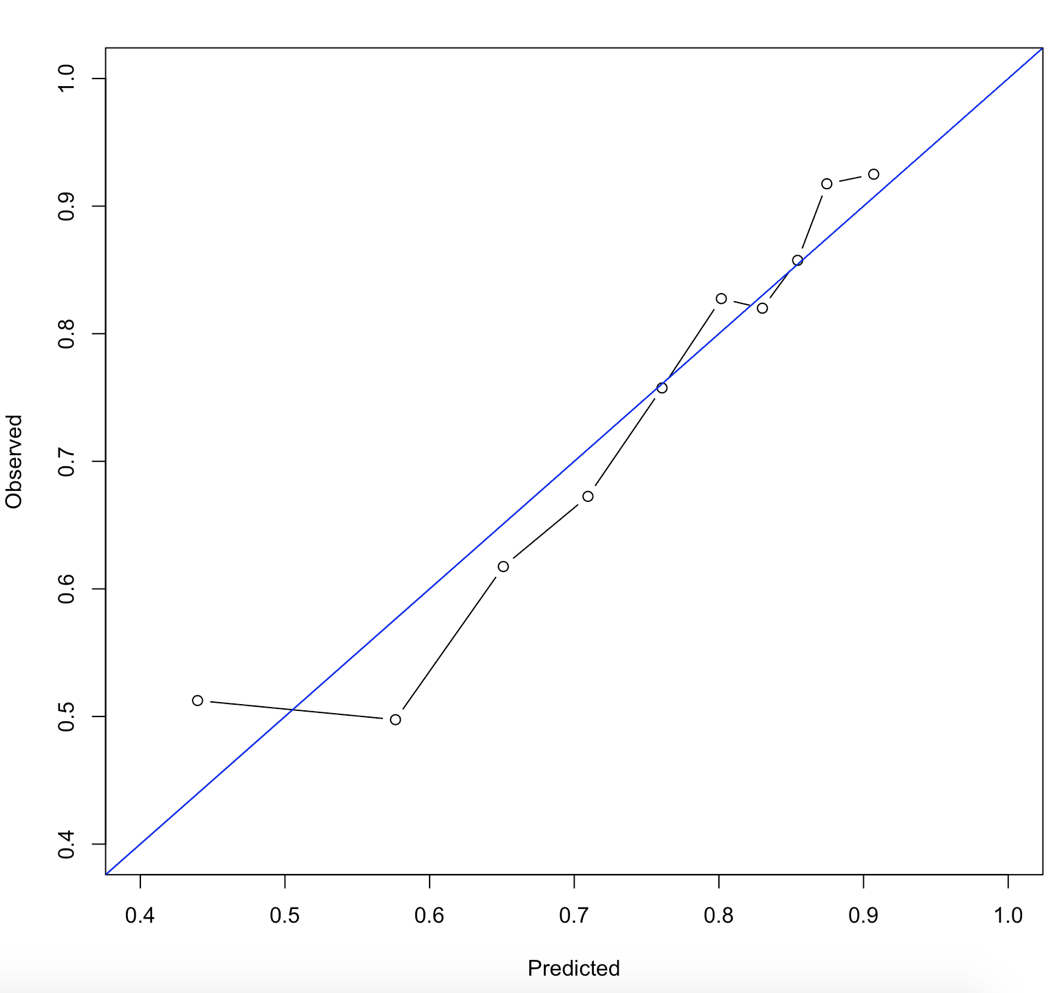
**

**Fig B.** Distribution of inverse probability of selection (IPS) weights


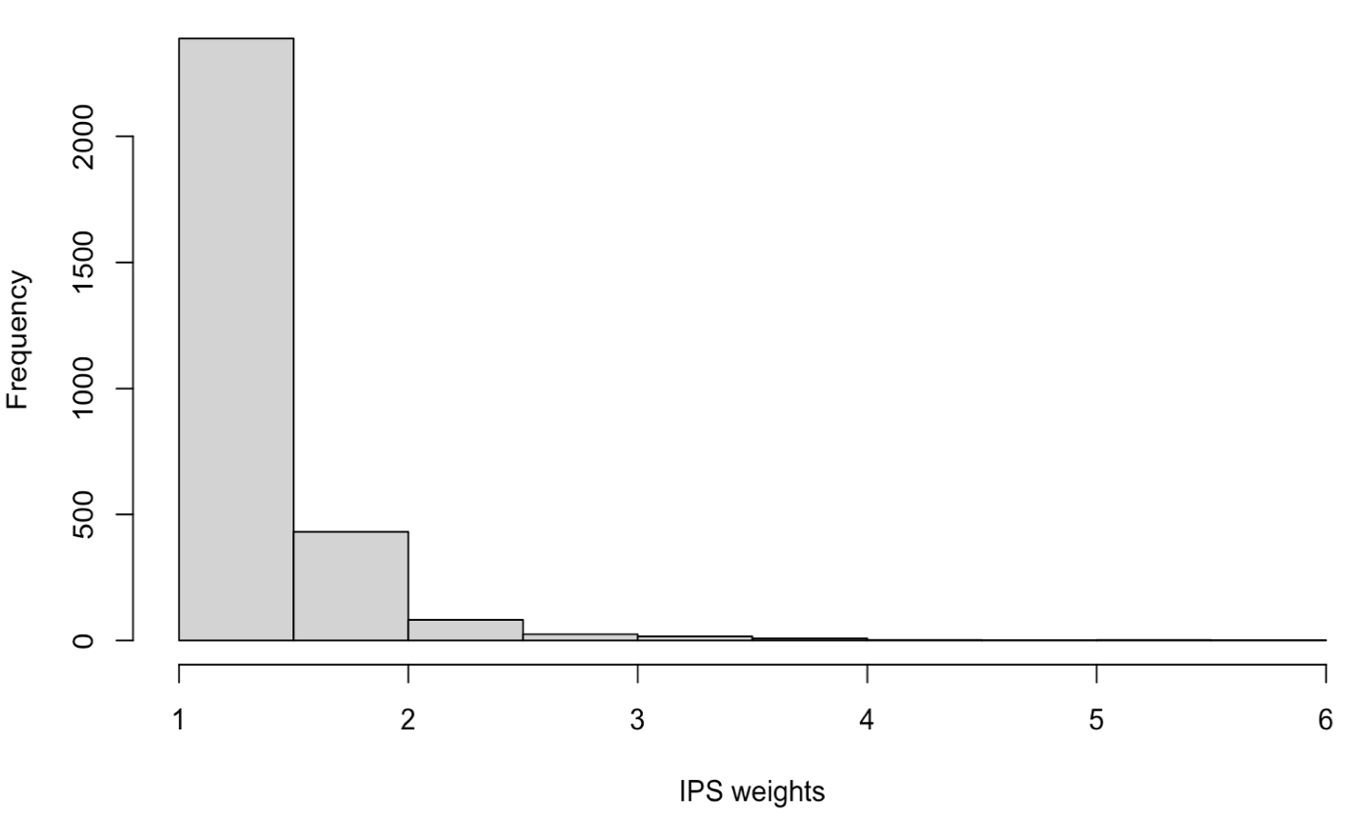


**Table A.** IPS weighted and unweighted cumulative hazard of death by treatment phase months

| Months of treatment | Unweighted | | | | | IPS Weighted | | | | |
| --- | --- | --- | --- | --- | --- | --- | --- | --- | --- | --- |
|  | **N at risk** | **Cumulative deaths** | **Cumulative hazard** | **95%CI** | | **N at risk** | **Cumulative deaths** | **Cumulative hazard** | **95%CI** | |
| 3 | 2867 | 102 | 3.50 | 2.79 | 4.11 | 3909 | 122 | 3.07 | 2.45 | 3.62 |
| 6 | 2722 | 144 | 4.98 | 4.10 | 5.66 | 3699 | 187 | 4.77 | 3.91 | 5.45 |
| 9 | 1258 | 179 | 7.01 | 5.80 | 7.79 | 1794 | 240 | 6.95 | 5.71 | 7.76 |
| 12 | 303 | 181 | 7.39 | 6.04 | 8.25 | 470 | 244 | 7.36 | 5.96 | 8.27 |
| 18 | 41 | 181 | 7.39 | 6.04 | 8.25 | 64 | 244 | 7.36 | 5.96 | 8.27 |
| 24 | 2 | 181 | 7.39 | 6.04 | 8.25 | 4 | 244 | 7.36 | 5.96 | 8.27 |

*IPS = inverse probability of selection*

**Table B**. IPS weighted and unweighted cumulative hazard of death by post-treatment phase months

| Months  post treatment | Unweighted | | | | | IPS Weighted | | | | |
| --- | --- | --- | --- | --- | --- | --- | --- | --- | --- | --- |
|  | **N at risk** | **Cumulative deaths** | **Cumulative hazard** | **95%CI** | | **N at risk** | **Cumulative deaths** | **Cumulative hazard** | **95%CI** | |
| 3 | 2753 | 8 | 0.29 | 0.09 | 0.49 | 3732 | 15 | 0.41 | 0.12 | 0.69 |
| 6 | 2722 | 10 | 0.36 | 0.14 | 0.59 | 3688 | 19 | 0.51 | 0.19 | 0.83 |
| 9 | 2560 | 19 | 0.71 | 0.39 | 1.02 | 3446 | 35 | 0.96 | 0.52 | 1.39 |
| 12 | 1642 | 22 | 0.87 | 0.50 | 1.23 | 2230 | 39 | 1.13 | 0.64 | 1.60 |
| 18 | 63 | 27 | 1.97 | 0.59 | 3.30 | 106 | 48 | 2.14 | 1.02 | 3.21 |
| 22 | 2 | 27 | 1.97 | 0.59 | 3.30 | 3 | 48 | 2.14 | 1.02 | 3.21 |

*IPS = inverse probability of selection*

**Equation B.** Cox proportional hazards model for in-treatment case fatality

$$\ln\left( h\left( t | X \right) \right)={\ln\left( h_{0}\left( t \right) \right)+ \beta}_{1}X_{sex}+\beta_{2}X_{age}+ \beta_{3}X_{SiteOfDisease}+ \beta_{4}X_{previousOrnew}+ \beta_{5}X_{residence}+ \beta_{6}X_{OutsideEnrolDist}+spline(adherence)$$

*Where: h(t) is the hazard over time, SiteofDisease = Extrapulmonary/pulmonary TB, Previous/new = previous or new TB case, Residence = Slum/non-slum residence, OutsideEnrolDist = Residential district outside PPSA enrolment district, adherence = proportion of doses taken.*

**Equation C.** Cox proportional hazards model for post-treatment case fatality

$$\ln\left( h\left( t | X \right) \right)={\ln\left( h_{0}\left( t \right) \right)+ \beta}_{1}X_{sex}+\beta_{2}X_{age:time}+ \beta_{3}X_{SiteOfDisease}+ \beta_{4}X_{previousOrnew}+ \beta_{5}X_{residence}+ \beta_{6}X_{OutsideEnrolDist}+\beta_{7}X_{adherence}$$

*Where: h(t) is the hazard over time and age: time is an interaction term between age (years) and time (post-treatment phase months). HRs for the interaction term were: 1.00, 95%CI: 0.99-1.00 in the weighted and unweighted models. SiteofDisease = Extrapulmonary/pulmonary TB, Previous/new = previous or new TB case, Residence = Slum/non-slum residence, OutsideEnrolDist = Residential district outside PPSA enrolment district, adherence = proportion of doses taken.*

Section 2: Secondary analysis

**Equation D** – Selection model (secondary analysis)

$logit\left( p\left( R=1 | X \right) \right)=\beta_{0}+\beta_{1}X_{age}+\beta_{2}X_{sex}+ \beta_{3}X_{SiteofDisease}+ \beta_{4}X_{PreviousOrNew}+ \beta_{5}X_{Residence}+ \beta_{6}X_{OutsideEnrolDist}+spline\left( tx\_duration \right){+ \beta}_{7}X_{adherence}+spline\left( since\_tx \right)+ \beta_{8}X_{HIV}+ \beta_{9}X_{Diabetes}+ \beta_{10}X_{After\_expansion}$ +

*Where: R = 1 = Responded to phone survey, SiteofDisease = Extrapulmonary/pulmonary TB, Previous/new = previous or new TB case, Residence = Slum/non-slum residence, OutsideEnrolDist = Residential district outside PPSA enrolment district, tx_duration = WHP-reported months of treatment duration, adherence = proportion of doses taken, since_tx = months since start of treatment and Nov 2022 (last month of phone survey), After_expansion = enrolled after PPSA program expansion (June 2020).*

**Fig C.** Hosmer-Lemeshow plot of selection model fit (observed vs predicted values) (secondary analysis):


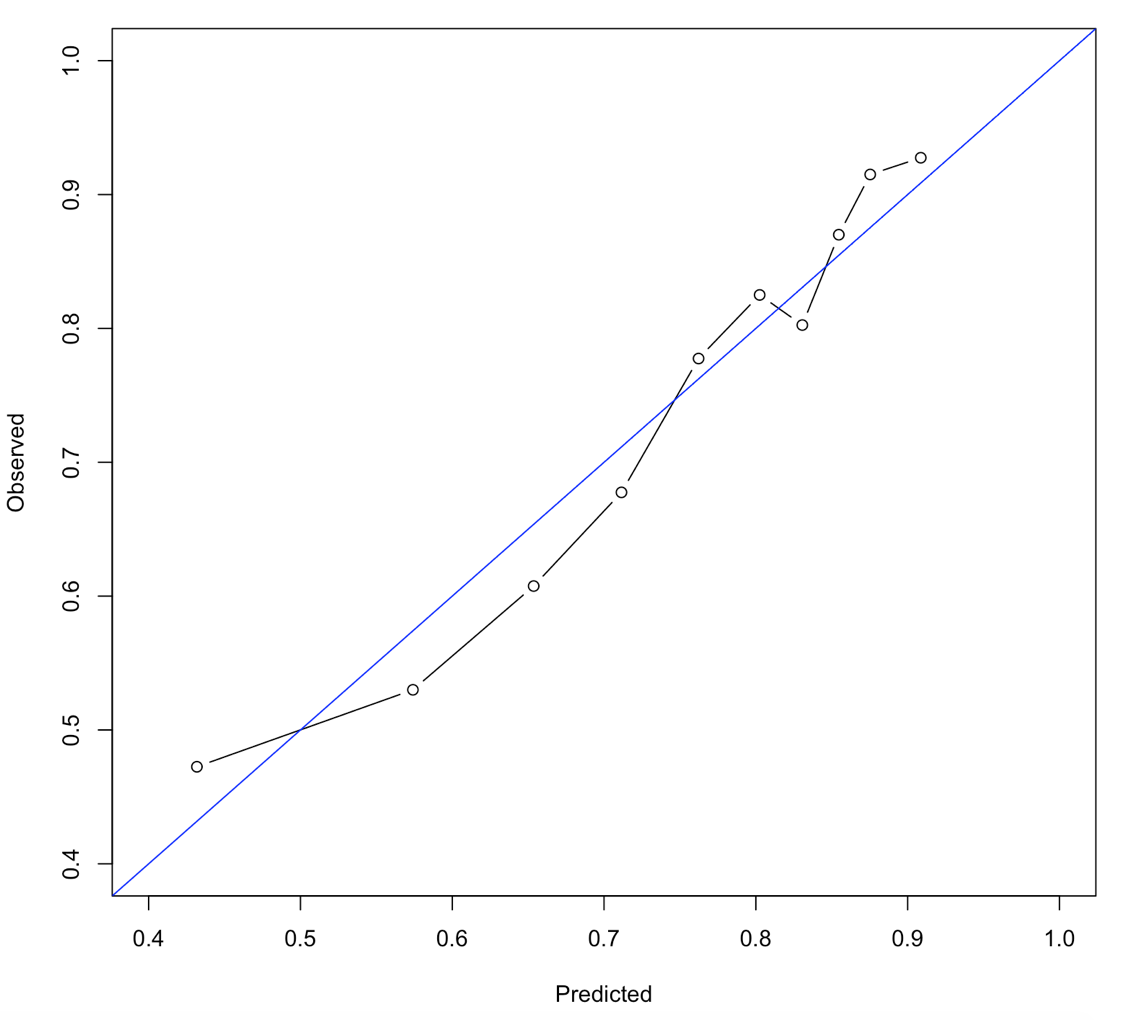


**Table C.** Covariate balance between full cohort and IPS weighted observed cohort (Mean [Variance]), using expanded selection model (Equation S4)

| Characteristic | Full cohort | Weighted observed cohort |
| --- | --- | --- |
| Age (years) | 39.00 (273.27) | 39.22 (275.98) |
| Sex (proportion male)^a^ | 0.61 (0.24) | 0.61 (0.24) |
| Proportion New TB (vs previous)^a^ | 0.98 (0.02) | 0.98 (0.02) |
| Proportion with pulmonary TB (vs. extrapulmonary) | 0.80 (0.16) | 0.80 (0.16) |
| Adherence (proportion of doses taken)^a^ | 0.79 (0.14) | 0.79 (0.14) |
| Proportion with non-slum residence (vs. slum residence)^a^ | 0.97 (0.03) | 0.97 (0.03) |
| WHP-reported months of treatment^a^ | 6.36 (3.23) | 6.35 (3.29) |
| Proportion with residential district outside of PPSA enrolment district | 0.21 (0.17) | 0.21 (0.17) |
| Proportion HIV positive ^a^ | 0.01 (0.01) | 0.01 (0.01) |
| Proportion with diabetes ^a^ | 0.07 (0.07) | 0.08 (0.07) |
| Proportion enrolled after program expansion | 0.94 (0.06) | 0.94 (0.06) |

*IPS = inverse probability of selection*

^a^ Imputed

**Equation E** – Treatment phase Cox proportional hazards model (secondary analysis)

$\ln\left( h\left( t | X \right) \right)={\ln\left( h_{0}\left( t \right) \right)+ \beta}_{1}X_{sex}+\beta_{2}X_{age}+ \beta_{3}X_{SiteOfDisease}+ \beta_{4}X_{previousOrnew}+ \beta_{5}X_{residence}+ \beta_{6}X_{OutsideEnrolDist}+spline\left( adherence \right)+ \beta_{7}X_{HIV}+ \beta_{8}X_{Diabetes}+ \beta_{9}X_{Smoking}$

*Where: h(t) is the hazard over time, SiteofDisease = Extrapulmonary/pulmonary TB, Previous/new = previous or new TB case, Residence = Slum/non-slum residence, OutsideEnrolDist = Residential district outside PPSA enrolment district, adherence = proportion of doses taken, smoking = current/past smoking (vs. never)*

**Table D.** Covariates associated with in-treatment case fatality among people with drug-susceptible TB in Bihar, India, in the first year of the COVID-19 pandemic (secondary analysis)

|  | Unweighted | | | IPS weighted | | |
| --- | --- | --- | --- | --- | --- | --- |
|  | **HR** | **95%CI** | | **HR** | **95%CI** | |
| Slum residence (Ref: Non-slum residence) | 1.06 | 0.26 | 2.08 | 1.16 | 0.27 | 2.22 |
| Previous TB (Ref: New TB) | 1.68 | 0.61 | 3.16 | 1.87 | 0.61 | 3.46 |
| Pulmonary TB (Ref: Extrapulmonary TB) | 1.16 | 0.82 | 1.76 | 1.17 | 0.81 | 1.78 |
| Age (years) | 1.04 | 1.04 | 1.05 | 1.04 | 1.03 | 1.05 |
| Sex, Male (Ref: Female) | 1.30 | 0.94 | 1.87 | 1.29 | 0.91 | 1.90 |
| District of residence outside PPSA enrolment district | 1.38 | 0.95 | 1.96 | 1.29 | 0.88 | 1.95 |
| HIV positive | 1.59 | 0.36 | 3.23 | 2.02 | 0.44 | 4.10 |
| Diabetic | 0.77 | 0.39 | 1.30 | 0.74 | 0.36 | 1.27 |
| Smoking (current or past, Ref: never) | 0.31 | 0.17 | 0.49 | 0.31 | 0.16 | 0.51 |

*IPS=inverse probability of selection.*

**Fig D.** Non-linear relationship between TB treatment adherence proportion and hazard of in-treatment death (secondary analysis)


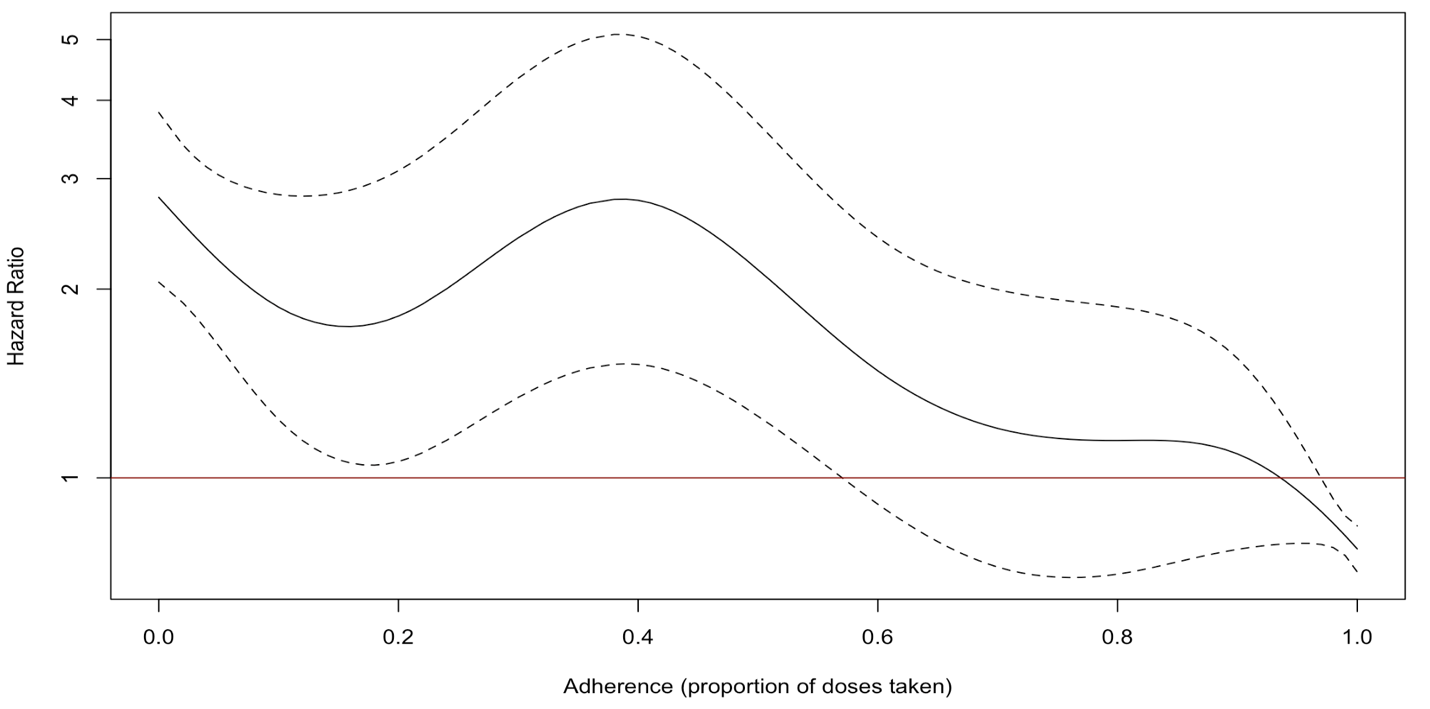

Supplement: S1 Text — (DOCX) [file pgph.0003277.s001.docx]
